# Distribution and Development of Peripheral Glial Cells in the Human Fetal Cochlea

**DOI:** 10.1371/journal.pone.0088066

**Published:** 2014-01-31

**Authors:** Heiko Locher, John C. M. J. de Groot, Liesbeth van Iperen, Margriet A. Huisman, Johan H. M. Frijns, Susana M. Chuva de Sousa Lopes

**Affiliations:** 1 Department of Anatomy and Embryology, Leiden University Medical Center, Leiden, the Netherlands; 2 Department of Otorhinolaryngology, Leiden University Medical Center, Leiden, the Netherlands; 3 Department for Reproductive Medicine, Ghent University Hospital, Ghent, Belgium; University of South Florida, United States of America

## Abstract

The adult human cochlea contains various types of peripheral glial cells that envelop or myelinate the three different domains of the spiral ganglion neurons: the central processes in the cochlear nerve, the cell bodies in the spiral ganglia, and the peripheral processes in the osseous spiral lamina. Little is known about the distribution, lineage separation and maturation of these peripheral glial cells in the human fetal cochlea. In the current study, we observed peripheral glial cells expressing SOX10, SOX9 and S100B as early as 9 weeks of gestation (W9) in all three neuronal domains. We propose that these cells are the common precursor to both mature Schwann cells and satellite glial cells. Additionally, the peripheral glial cells located along the peripheral processes expressed NGFR, indicating a phenotype distinct from the peripheral glial cells located along the central processes. From W12, the spiral ganglion was gradually populated by satellite glial cells in a spatiotemporal gradient. In the cochlear nerve, radial sorting was accomplished by W22 and myelination started prior to myelination of the peripheral processes. The developmental dynamics of the peripheral glial cells in the human fetal cochlea is in support of a neural crest origin. Our study provides the first overview of the distribution and maturation of peripheral glial cells in the human fetal cochlea from W9 to W22.

## Introduction

Schwann cells, the major type of peripheral glial cells (PGCs), envelop and/or myelinate the spiral ganglion neurons (SGNs) in the cochlea and are essential to normal hearing. Demyelinating diseases of the peripheral nervous system result in differences in the velocity of action potential propagation between individual nerve processes [1]. Depending on the degree of demyelination, this loss of neural synchrony leads to moderate sensorineural hearing loss or, if there is a complete conduction block, to deafness [2]–[4]. One major peripheral neuropathy affecting hearing is Charcot-Marie-Tooth disease, a genetically and clinically heterogeneous group of disorders which includes mutations in genes that are involved in myelination [5]–[8]. Other causes of demyelination of peripheral nerves, and hence potentially leading to sensorineural hearing loss, include autoimmune diseases such as the Guillain-Barré syndrome, and infectious diseases such as leprosy [9]–[12]. Loss of myelin may also be involved in the development of age-related sensorineural hearing loss [13].

**Figure 1:**
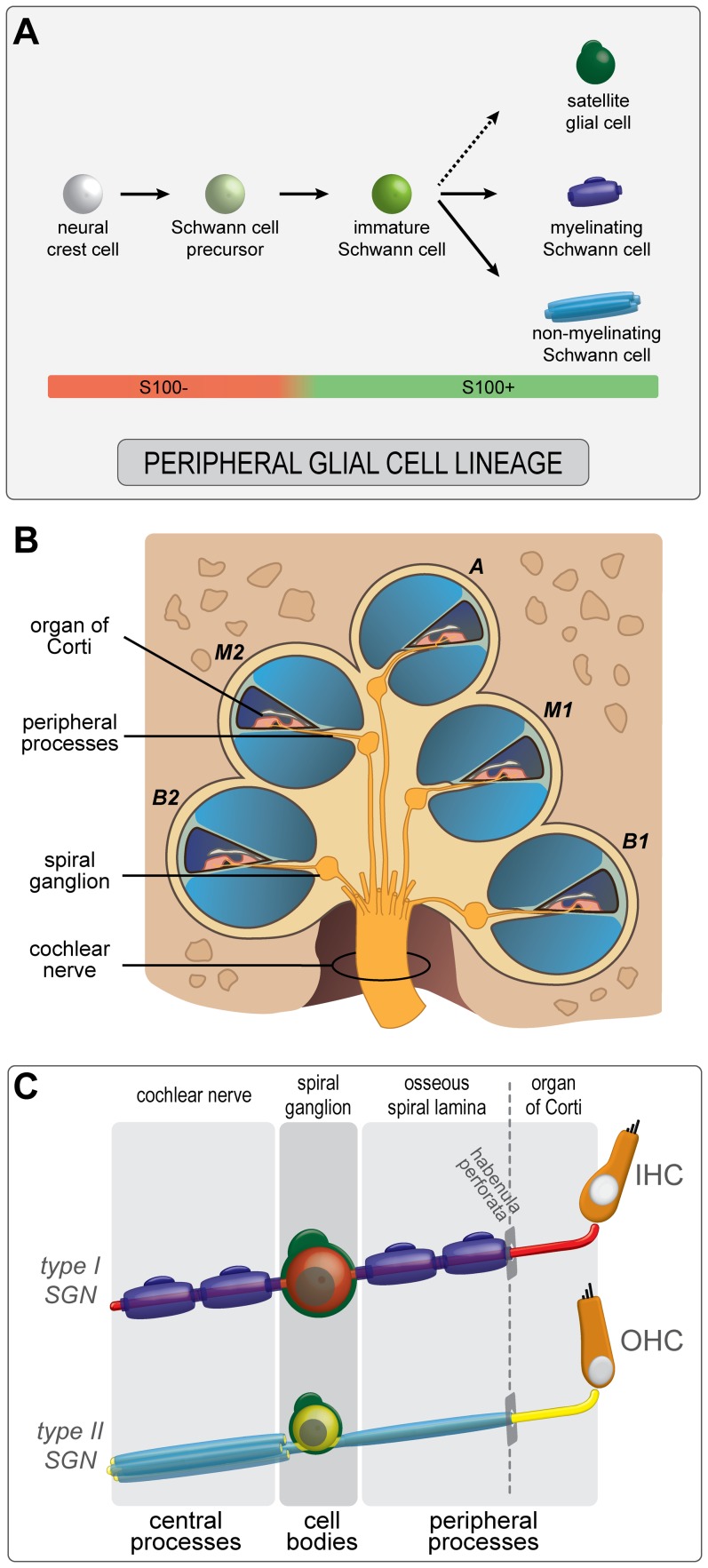
Capturing PGCs in the human cochlea. (A) Schematic model of PGC development in the human fetal cochlea. Neural crest cells differentiate via a Schwann cell precursor stage into S100+ immature Schwann cells. The immature Schwann cells subsequently maturate into myelinating and non-myelinating Schwann cells, and (presumably) satellite glial cells. (B) Schematic illustration of a mid-modiolar cut of the adult human cochlea, showing the lower basal turn (B1), upper basal turn (B2), lower middle turn (M1), upper middle turn (M2) and the apex (A). (C) Schematic illustration of the PGCs in the adult human cochlea. Satellite glial cells (green) envelop all SGN cell bodies. Non-myelinating Schwann cells (light blue) ensheath both the central and peripheral processes of the type II SGNs (yellow) that innervate the outer hair cells (OHC). Myelinating Schwann cells (dark blue) ensheath and myelinate both processes of the type I SGNs (red) that innervate the inner hair cells (IHC). Beyond the habenula perforata, in the organ of Corti, neither Schwann cell types ensheath the most distal part of the peripheral processes of type I and type II SGNs.

Based on animal studies, it is commonly accepted that all PGCs derive from the neural crest and migrate along peripheral nerves to their target locations [14], [15]. There, Schwann cell precursors become immature Schwann cells, which subsequently differentiate into myelinating or non-myelinating Schwann cell phenotypes (Fig. 1A). Individual processes of peripheral neurons are singled out by pro-myelinating Schwann cells in a process known as radial sorting. Once ensheathment is completed, those Schwann cells will start to produce myelin, becoming myelinating Schwann cells [14]. The myelin sheath consists of multiple layers of tightly packed myelin surrounding individual nerve processes and functions to increase axonal conduction velocity [16]. Non-myelinating Schwann cells will envelop numerous unmyelinated neuronal processes, forming the so-called Remak bundles in which the individual nerve processes remain separated by cytoplasmic extensions of the non-myelinating Schwann cell [17], [18]. Although Schwann cell differentiation has been investigated extensively, less is known about the development of a third type of PGCs, satellite glial cells. Satellite glial cells are thought to play a role in the microenvironment, protecting, supporting and communicating with the neuronal cell bodies [19], [20]. Avian studies suggest that satellite glial cells and mature Schwann cells derive from a common precursor cell expressing the marker S100 [21] (Fig. 1A). The differentiation cascade that leads to the formation of satellite glial cells in humans remains to be investigated.

In the adult human cochlea, all three PGC types are intimately associated with SGNs. SGNs are bipolar or pseudo-unipolar neurons that transmit electrical signals encoding sound from cochlear hair cells to the brain. They are usually classified as type I SGNs (90–95% of the total population) and type II (5–10%) SGNs. Both processes of the bipolar type I SGNs, the central process in the cochlear nerve (CN) and the peripheral process in the osseous spiral lamina (Fig. 1B–C), are enwrapped in myelin sheaths that are produced by myelinating Schwann cells [22], [23]. The second type of PGCs, the non-myelinating Schwann cells (Fig. 1C), ensheath but do not myelinate the central and peripheral processes of the type II SGNs [24], [25]. Neither type of Schwann cells ensheath the peripheral processes (that innervate the cochlear hair cells in the organ of Corti) beyond the habenula perforata [26]. In the spiral ganglion (SG), the cell bodies of the SGNs are enveloped by the third type of PGCs, the satellite glial cells (Fig. 1C). In contrast to most mammalian species, satellite glial cells in the human SG seldom myelinate the SGN cell bodies [13], [22], [27], [28].

PGCs have been observed using light microscopy in the human SG and along the central processes of SGNs from 11 weeks of gestation (W11, i.e. 9 weeks of fetal development) onwards [29]. Other reports have looked at earlier stages, but failed to discriminate between SGNs and PGCs in the SG [30]. Using electron-microscopy, it appeared that myelination of the central processes of the SGNs in the CN in humans starts at W20 [31]. At the peripheral processes, a study using Woelcke's iron-hematoxylin method [32] suggested that myelination starts in at W22, although transmission electron microscopy suggested that it commences at W24 [33]. However, it cannot be excluded that the onset of myelination of the peripheral processes is at an earlier stage, especially since younger specimens were not available to these authors.

In the present study, we investigated the distribution and maturation of PGCs in the human fetal cochlea from W9 to W22. Using immunohistochemistry, we observed PGCs in the cochlea as early as W9 and we defined their developmental phenotype in the three domains of the SGNs: (1) their central processes, (2) their cell bodies, and (3) their peripheral processes. Studies on Schwann cell development are not only important to investigate the onset of myelination (which is one indicator for the maturation of the auditory system and, hence, related to the onset of hearing), but are also needed to better understand the relationship of Schwann cells to auditory neuropathies, whether it be congenital disorders or disorders acquired during adult life.

## Materials and Methods

### Ethics statement

The use of human fetal material for this study was approved by the Medical Ethical Committee of the Leiden University Medical Center (protocol 08.087) and informed written consent was obtained in accordance with the WMA Declaration of Helsinki guidelines.

### Human fetal cochleae

Twenty-four fetal cochleas were isolated from human fetal material collected after elective abortion (using vacuum aspiration). Gestational age (in weeks and days) was determined using obstetric ultrasonography (W9, n = 1; W10, n = 2; W11, n = 1; W12, n = 2; W14, n = 2; W15, n = 2; W16, n = 2; W17, n = 3; W18, n = 5; W19, n = 2; W20, n = 1; W22, n = 1). The cochleas were isolated in PBS, fixed in 4% paraformaldehyde in PBS overnight at 4°C, decalcified and embedded in paraffin as previously described [34].

### Immunofluorescence

The cochleas were cut (5 µm sections) in the sagittal plane using a RM2255 microtome (Leica). Sections were deparaffinized using standard procedures and immunohistochemistry was performed as previously described [34]. Briefly, sections were treated 12 minutes at 97°C with 0.01M sodium citrate buffer (pH 6.0) for antigen retrieval, blocked with 1% bovine serum albumin (Sigma-Aldrich) in PBS containing 0.05% Tween-20 (Promega), and consecutively incubated with primary and secondary antibodies diluted in blocking solution. Nuclei were stained with 4′,6-diamidino-2-phenylindole (DAPI, Vector Laboratories). The primary antibodies used in this study were rabbit anti-myelin basic protein (MBP, 1∶4000, A0623, DAKO), mouse anti-myosin VIIa (MYO7A, 1∶40, 138-1 supernatant, DSHB), rabbit anti-nerve growth factor receptor (NGFR, 1∶200, 07-476, Millipore), chicken anti-peripherin (PRPH, 1∶200, ab39374, Abcam), rabbit anti-S100 calcium binding protein B (S100B, 1∶200, ab52642, Abcam), rabbit anti-SOX9 (1∶200, ab5535, Millipore), goat anti-SOX10 (1∶50, sc-17342, Santa Cruz), and mouse anti-class III β-tubulin (TUBB3, 1∶200, ab78078, Abcam). The secondary antibodies used were Alexa Fluor (AF) conjugated immunoglobulins (Life Technologies): AF 488 donkey anti-mouse (A-21202), AF 488 donkey anti-rabbit (A-21206), AF 488 donkey anti-goat (A-11055), AF 488 goat anti-chicken (A-11039), AF 568 donkey anti-mouse (A-10037), AF 568 donkey anti-rabbit (A-10042), AF 647 donkey anti-mouse (A-31571), AF 647 donkey anti-rabbit (A-31573) and AF 647 donkey anti-goat (A-21447), all at 1∶500 dilution. As antibody specificity controls, primary antibodies were omitted and rabbit immunoglobulin fraction (X0903, DAKO) was used to confirm that the cytoplasmic fluorescence observed in all SGN cell bodies when using higher concentrations of the MBP-antibody was non-specific.

### Image acquisition and processing

Confocal images were made with either a Leica TCS SP5 inverted or a Leica SP8 upright microscope, operating under the Leica Application Suite Advanced Fluorescence software (LAS AF) using Leica objectives (20×/0.5 dry HCX PL Fluotar; 40×/1.3 oil HC PL Apo; 63×/1.4 oil HC PL Apo; 100×/1.4 oil HCX PL Fluotar). Maximal projections were obtained from image stacks that were generated by scanning sections throughout their full depth with z-steps of 0.5 µm, or with a sampling density according to the Nyquist rate when image restoration (deconvolution) was applied using Huygens Professional version 4.3.1 software (Scientific Volume Imaging). Brightness and contrast adjustments consistent with image manipulation policies were performed either with LAS AF, ImageJ version 1.47a (National Institutes of Health, http://imagej.nih.gov/ij) or Adobe Photoshop CS6 (Adobe Systems) image-processing software.

## Results

### Human W9 fetal cochlea contains SOX9+/SOX10+/S100B+ PGCs

**Figure 2:**
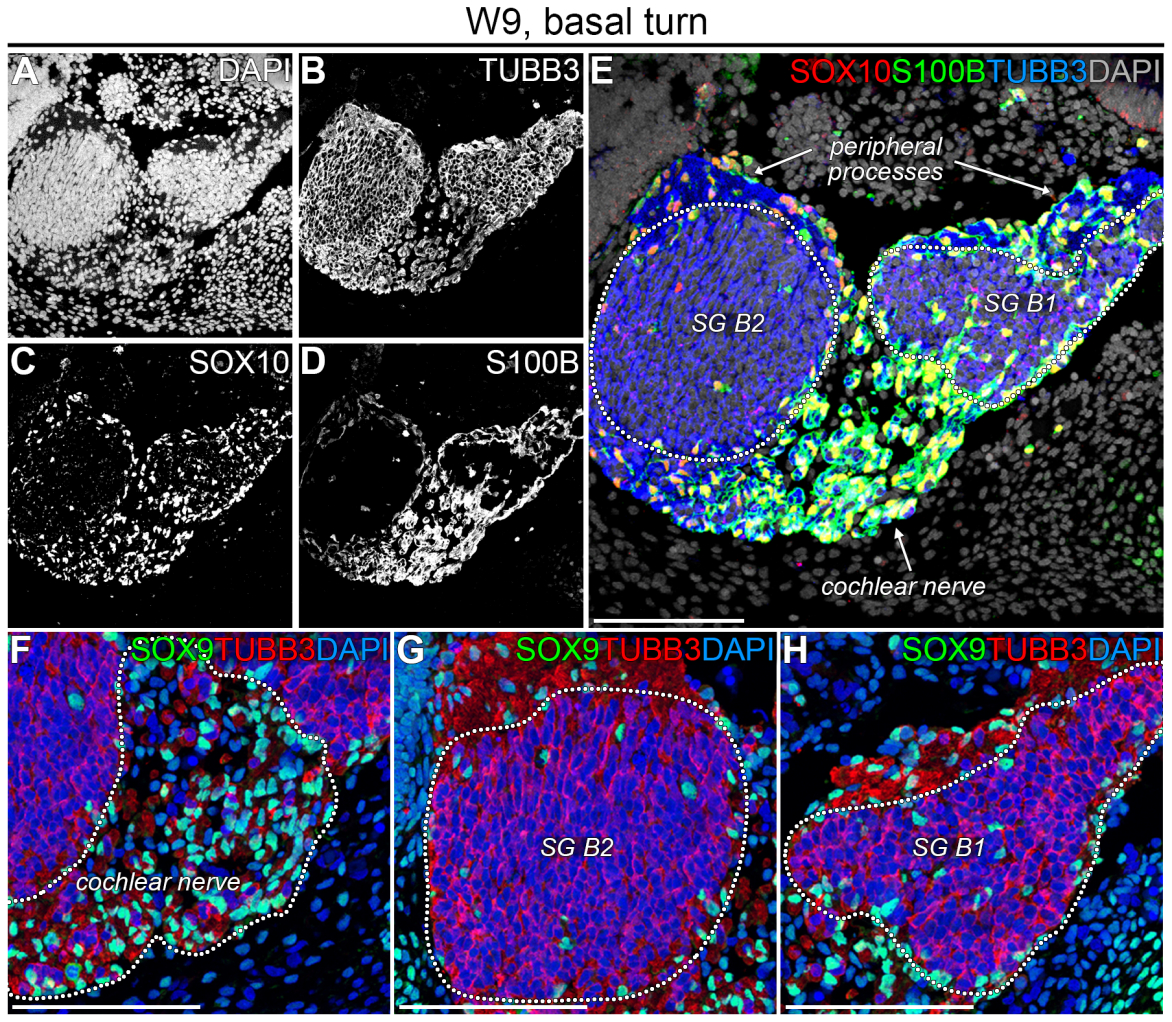
PGCs expressed SOX10, SOX9 and S100B in the W9 human fetal cochlea. (A–E) Confocal images of a cochlea at W9 showing DAPI (A), TUBB3 (B), SOX10 (C), and S100B (D) and the merged image (E). The spiral ganglion is delineated by the dotted line. (F–H) Confocal images of a cochlea at W9 showing the cochlear nerve (F), the spiral ganglion in the upper basal turn (G), and the spiral ganglion in the lower basal turn (H), delineated with dotted lines and immunostained with antibodies against SOX9 and TUBB3. Cell nuclei were visualized with DAPI. Abbreviations: B1, lower basal turn; B2, upper basal turn; SG, spiral ganglion. Scale bar = 100 µm.

To our knowledge, the presence of PGCs has not been reported in human fetal cochleas younger than W11 [29]. Here, we have investigated the distribution and phenotype of PGCs at W9, the youngest cochlear specimen we were able to obtain. At W9, the cochlear duct consisted of one full turn, the future basal turn. To identify PGCs, we immunostained for SOX10 (a nuclear marker for PGCs regardless of developmental stage [35], [36]), S100B (a cytoplasmic marker expressed in animals by immature and mature Schwann cells but not by Schwann cell precursors [37], [38]) and TUBB3 (a general marker of SGNs) (Fig. 2A–H). In the CN, abundant SOX10+/S100B+ PGCs were detected surrounding essentially all central processes (Fig. 2E). SOX10+/S100B+ PGCs were also observed at the peripheral processes in the lower (B1) and the upper (B2) basal turn of the SG (Fig. 2E). In the SG, we identified SOX10+/S100B+ PGCs mainly along its edge and only a few in its center (Fig. 2E). Immunostaining for SOX9, a nuclear neural crest and PGC marker [39], [40], revealed a similar pattern of expression to SOX10 and S100B in PGCs (Fig. 2F–H), indicating that at W9 the majority of PGCs located along the neuronal processes have passed the SOX9+/SOX10+/S100B− progenitor phase and have developed into a more mature SOX9+/SOX10+/S100B+ phenotype. Based on their distribution density, these PGCs most likely migrate from the central processes to the peripheral processes along the periphery of the SG and may start colonizing the SG giving rise to the satellite glial cells.

### SOX9+/SOX10+/S100B+ PGCs invade the SG in a spatiotemporal gradient

The developing human cochlea is characterized by distinct temporal and a spatial gradients at various levels including hair cell development, organ of Corti maturation, and hair cell innervation [34]. Therefore, we investigated whether PGCs in the SG displayed a similar developmental pattern within the cochlea.

**Figure 3:**
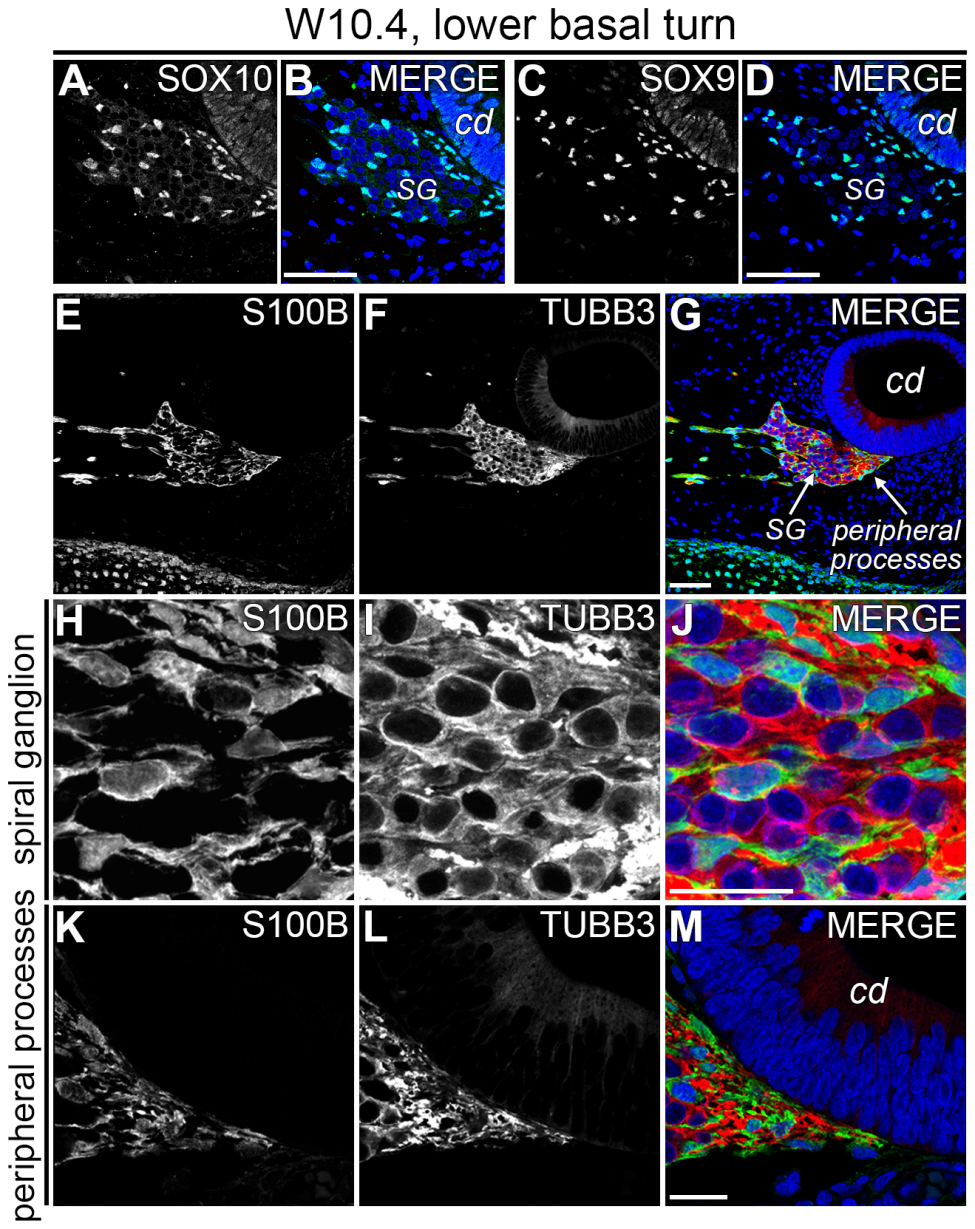
PGCs expressed SOX10, SOX9 and S100B in the W10.4 human fetal cochlea. (A–B) Confocal images of the lower basal turn of a W10.4 cochlea immunostained for SOX10 and SOX10 merged with DAPI. (C–D) Confocal images of an adjacent section immunostained for SOX9 and SOX9 merged with DAPI. (E–G) Confocal images of the lower basal turn of a W10.4 cochlea immunostained for S100B (E) and TUBB3 (F) and the merged image with DAPI (G). (H–J) High-magnification view of the center of the spiral ganglion. (K–M) Detail of the peripheral processes at their distal end. Abbreviations: cd, cochlear duct; SG, spiral ganglion. Scale bar = 50 µm (A–G) or 20 µm (H–M).

**Figure 4:**
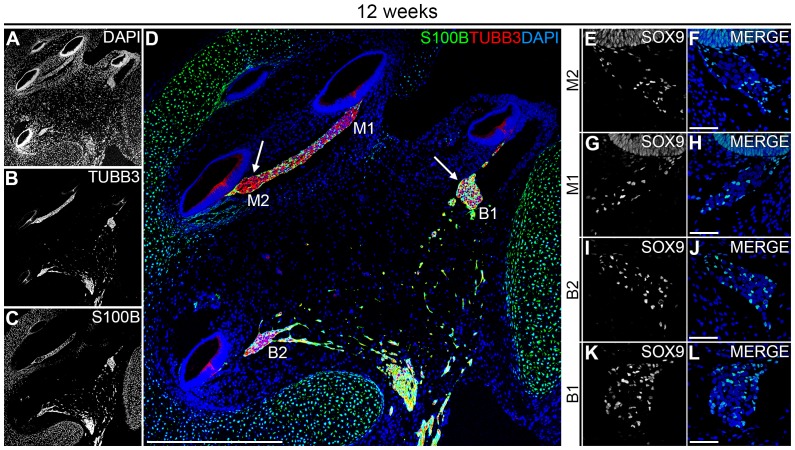
A spatial gradient of S100+/SOX9+ PGCs in the W12 human fetal cochlea. (A–D) Confocal images of a cochlea at W12 showing DAPI (A) TUBB3 (B) and S100B (C) and the merged image (D). In E, the left arrow points to the spiral ganglion in the upper middle turn, whereas the right arrow points to the spiral ganglion in the lower basal turn. (E–L) Confocal images of a cochlea at W12 immunostained for SOX9 and SOX9 merged with DAPI showing the spiral ganglion at M2 (E–F), M1 (G–H), B2 (I–J) and B1 (K–L). Abbreviations: B1, lower basal turn; B2, upper basal turn; M1, lower middle turn; M2, upper middle turn. Scale bar = 500 µm (A–D) or 50 µm (E–L).

In the lower basal turn (B1) between W9 and W10.4, we observed an increased number of PGCs in the center of the SG (Fig. 2C–J and Fig. 3A–G), in agreement with our hypothesis that SOX9+/SOX10+/S100B+ PGCs gradually colonize the SG, there differentiating into satellite glial cells. At a higher magnification, the central region of the SG showed the formation of a network of PGCs between the cell bodies of the SGNs (Fig. 3H–J). However, PGCs at this stage (W10.4) did not yet cluster with the cell bodies of the SGNs. S100B+ PGCs were present along the peripheral processes as far as the most distal tips of those processes, right underneath the prosensory domain of the cochlear duct (Fig. 3K–M). In addition to the SGNs, the epithelium of the cochlear duct also weakly immunostained for TUBB3, with the brightest fluorescence in the prosensory domain (Fig. 3F and 4B).

By W12, dense immunostaining for S100B was observed in the lower basal turn (B1) of the SG (Fig. 4A–D, right arrow in D), suggesting the presence of many PGCs. In addition, a spatial gradient was visible in a basal-to-apical direction. From B1 to the upper middle turn (M2), S100B immunostaining was progressively less prominent in the center of the SG and PGCs were primarily present at the edge of the SG in M2 (Fig. 4A–D, left arrow in D). In agreement, immunostaining for SOX9 at W12 confirmed this basal to apical distribution of PGCs (Fig. 4E–L). We concluded that, similar to other aspects of cochlear development, PGCs also show distinct temporal and spatial gradients in colonizing the SG of the human fetal cochlea.

### PGCs envelope SGN cell bodies between W12 and W14

**Figure 5:**
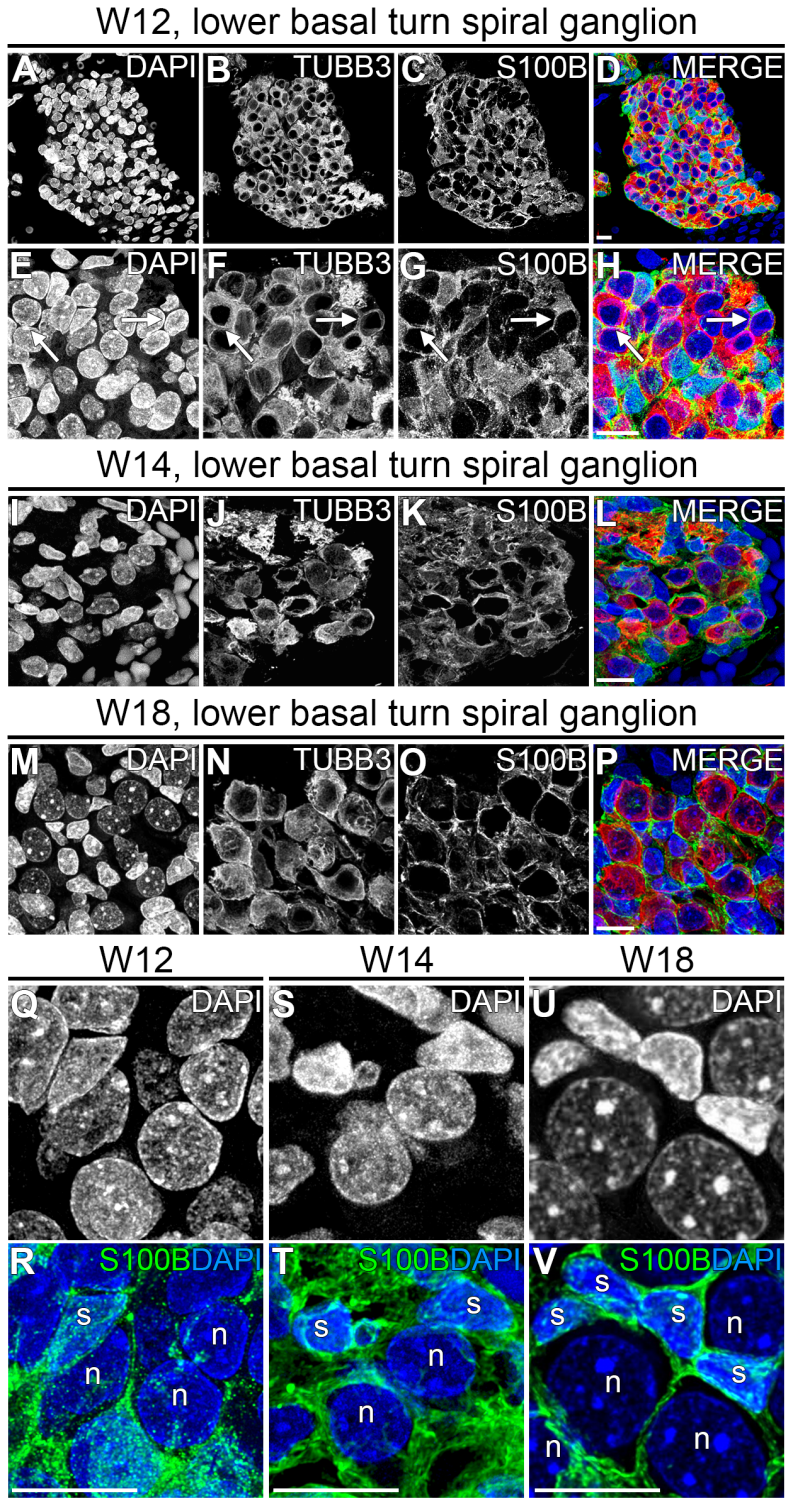
Development of satellite glial cells in the spiral ganglion. (A–D) Deconvoluted confocal images of the spiral ganglion in the lower basal turn at W12 showing DAPI (A, blue), TUBB3 (B, red), S100B (C, green) and the merged image (D). (E–H) High-magnification view of the spiral ganglion. Arrows point to SGN cell bodies that are enwrapped by S100B+ satellite glial cells. (I–L) Deconvoluted confocal images of the spiral ganglion in the lower basal turn at W14 showing DAPI (I, blue), TUBB3 (J, red), S100B (K, green) and the merged image (L). (M–P) Deconvoluted confocal images of the spiral ganglion in the lower basal turn at W18 showing DAPI (M, blue), TUBB3 (N, red), S100B (O, green), and the merged image (P). (Q–V) High-magnification view of cell nuclei (DAPI) and S100B+ satellite glial cells within the spiral ganglion at W12 (Q–R), W14 (S–T) and W18 (U–V). Abbreviations: s, satellite glial cell; n, spiral ganglion neuron. Scale bar = 10 µm.

The onset of envelopment of the cell bodies of the TUBB3+ SGNs by the S100B+ PGCs was observed at W12 in the basal turn of the SG (Fig. 5A–H, arrows) and by W14 the cell bodies of all SGNs in the SG were fully enveloped by S100B+ PGCs (Fig. 5I–L). By W18, this had become even more pronounced and S100B+ PGCs had become intimately associated with the SGNs (Fig. 5M–P).

**Figure 6:**
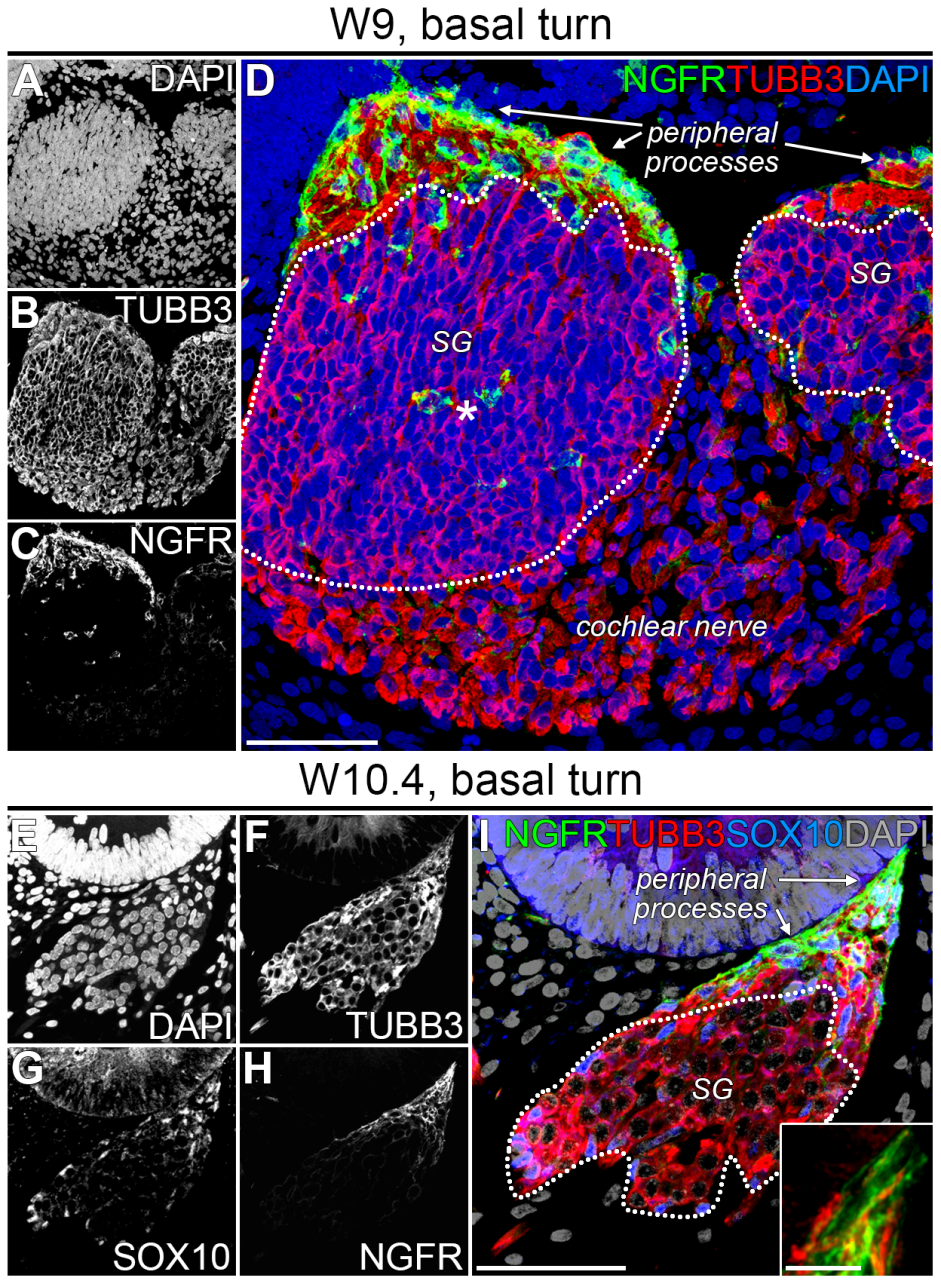
Immature Schwann cells along the peripheral processes express NGFR. (A–D) Confocal images of the lower basal turn of a cochlea at W9 showing DAPI (A), TUBB3 (B) and NGFR (C) and the merged image (D). The spiral ganglion is delineated by the dotted line. The asterisk marks two NGFR+ cells in the center of the spiral ganglion. (E–I) Confocal images of the lower basal turn of a cochlea at W10.4 showing DAPI (E), TUBB3 (F), SOX10 (G) and NGFR (H) and the merged image (I). The spiral ganglion is delineated by the dotted line. The inset shows a deconvoluted, high-magnification view of TUBB3 and NGFR at the distal tips of the peripheral processes. Abbreviations: SG, spiral ganglion. Scale bar = 50 µm or 5 µm (inset in I).

During the process of envelopment, we also noticed changes in the PGC nuclei. At W12, DAPI staining showed that SGN nuclei were generally round whereas the nuclei of the enveloping PGCs tended to be more angular or crescent shaped (Fig. 6Q–R). At W14, this difference became more prominent. In addition, PGC nuclei started to get organized around and facing the cell bodies of the SGNs and exhibited bright yet diffuse DAPI staining (Fig. 6S–T). By W18, the differences between the PGC nuclei and SGN nuclei were even more pronounced, with round SGN nuclei containing prominent nucleoli (Fig. 6U–V), much like the morphology in adults. At W18, each SGN cell body was surrounded by one or more PGCs.

### At W9, NGFR is expressed in PGCs along the peripheral processes

The (low affinity) nerve growth factor receptor (NGFR, also known as p75^NTR^) is expressed by Schwann cells at various locations within the adult human cochlea [41] and is expressed in mice and rats throughout the neural crest and Schwann cell lineages [14]. Immunostaining for NGFR at W9 and W10.4 revealed two different PGC phenotypes in the developing human cochlea. In the lower basal turn (B1), PGCs along the peripheral SGN processes strongly expressed NGFR both at W9 (Fig. 6A–D) and W10.4 (Fig. 6E–I). NGFR immunostaining could even be detected along the most distal tips of the peripheral processes just underneath the cochlear duct (Fig. 6I, inset). In contrast, PGCs located at the central processes in the CN did not express NGFR (Fig. 6A–D). In addition, a few NGFR+ cells were detected in the SG at W9 (Fig. 6A–D, asterisk in D), but not at W10.4 (Fig. 6E–I) or later stages.

### S100B+/NGFR+ PGCs cells do not penetrate the cochlear duct epithelium

**Figure 7:**
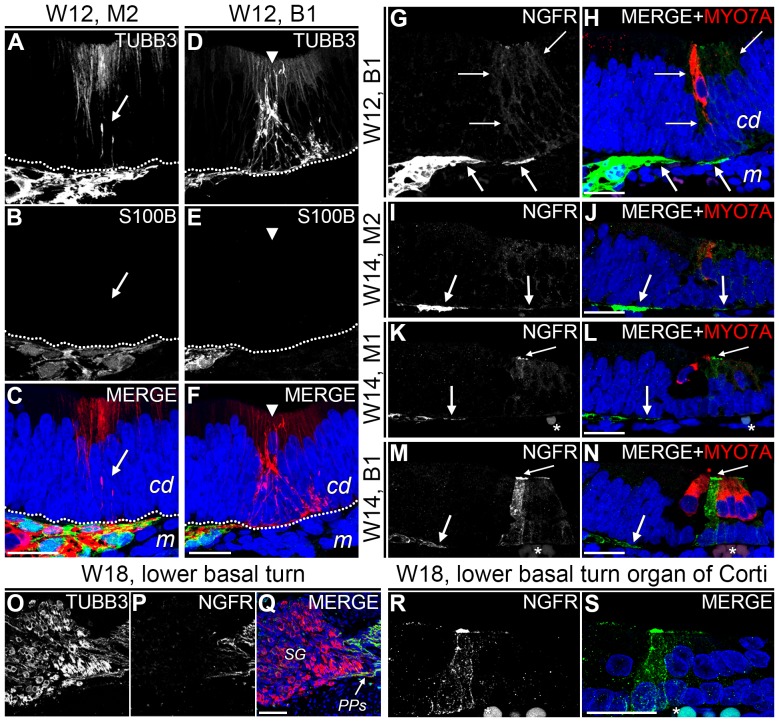
NGFR expression in the cochlear duct epithelium. (A–C) Confocal images of the cochlear duct epithelium in the upper middle turn of a cochlea at W12 showing TUBB3 (A, red), S100B (B, green) and the merged image with DAPI (C). The arrow points to penetrating TUBB3+ peripheral processes. (D–F) Confocal images of the cochlear duct epithelium in the lower basal turn showing TUBB3 (D, red), S100B (E, green) and the merged image with DAPI (F). The arrowhead points to the first developing (inner) hair cell. (G–H) Confocal images of the cochlear duct epithelium of the lower basal turn of a cochlea at W12 showing NGFR (G, green) and the merged image with DAPI (H) and MYO7A (red). The bold arrows point to the NGFR+ Schwann cells. The thin arrows outline the epithelial cells that weakly express NGFR. (I–J) Upper middle turn of a cochlea at W14 showing NGFR (I, green) and the merged image (J) with DAPI (blue) and MYO7A (red). The bold arrows point to the NGFR+ Schwann cells. (K–L) Lower middle turn of a W14 cochlea immunostained for NGFR (K, green) and the merged image with DAPI (blue) and MYO7A (red). The bold arrow points to the NGFR+ Schwann cells. The thin arrow points to a bright band of NGFR. (M–N) Lower basal turn of a W14 cochlea immunostained for NGFR (M, green) and the merged image (N) with DAPI (blue) and MYO7A (red). The bold arrows point to the NGFR+ Schwann cells. The thin arrow points to a band brightly immunostained for NGFR. (O–Q) Confocal images of the spiral ganglion in the lower basal turn of a cochlea at W18 showing TUBB3 (O), NGFR (P, green) and the merged image with DAPI (Q). (R–S) Confocal images of the organ of Corti of a cochlea at W18 showing NGFR (R, green) and the merged image with DAPI (S). Abbreviations: cd, cochlear duct; m, mesenchyme; B1, lower basal turn; M1, lower middle turn; M2, upper middle turn; SG, spiral ganglion; PPs, peripheral processes. * = autofluorescence of erythrocytes. Scale bar = 20 µm (A–N, R–S) or 50 µm (O–Q).

Since we consistently observed PGCs at the distal tips of the outgrowing peripheral processes (Fig. 3K–M and the inset in Fig. 6I), we investigated whether they would initially follow the peripheral processes into the cochlear duct epithelium, although Schwann cells are known to be absent in the mature organ of Corti [26]. We have previously shown that penetration of peripheral processes into the cochlear duct epithelium is observed in all turns at W12 and that they targeted and innervated the developing (future) inner hair cells [34]. At W12 in the upper middle turn (M2), multiple S100+ PGCs were located directly outside the cochlear duct epithelium, and although some TUBB3+ neurites could be seen penetrating into the prosensory domain, S100B expression could not be observed beyond this border (Fig. 7A–C). In the lower basal turn (B1), many peripheral processes were found targeting the first developing hair cell within the cochlear duct epithelium (Fig. 7D–F, arrowhead). Here, S100B+ PGCs did not extend into the cochlear duct epithelium either. Immunostaining for NGFR confirmed these results (Fig. 7G–H, bold arrows). Expression of NGFR was also evident directly underneath the cochlear duct epithelium at W14, both in the basal and middle turns (Fig. 7I–N, bold arrows). Together, these findings indicate that PGCs do not follow the peripheral processes into the cochlear duct epithelium during development.

### NGFR is expressed by pillar cells within the developing human organ of Corti

Interestingly, just after the onset of the first hair cell differentiation at B1 of W12, we also observed weak NGFR expression within the cochlear duct epithelium in a group of cells located around the (future) inner hair cell, marked by MYO7A expression (Fig. 7G–H, thin arrows). At W14, similar weak NGFR expression was observed at M2 (Fig. 7I–J). At M1, expression was increased in the cell lateral to the inner hair cell, with a bright band at its apical surface, corresponding to the cuticular plate (Fig. 7K–L, thin arrows). At W14, the outer hair cells had also differentiated at B1 and strong NGFR expression was observed in the putative outer pillar cell (Fig. 7M–N, thin arrows). In contrast to the observed NGFR expression, none of the turns at W12–W14 showed any S100B expression in the developing organ of Corti (data not shown). In the subsequent weeks (i.e., up to W18), similar NGFR expression patterns were observed both in the PGCs along the peripheral processes (Fig. 7O–Q) and within the organ of Corti (Fig. 7R–S).

### Myelination has started in the W22 cochlear nerve

**Figure 8:**
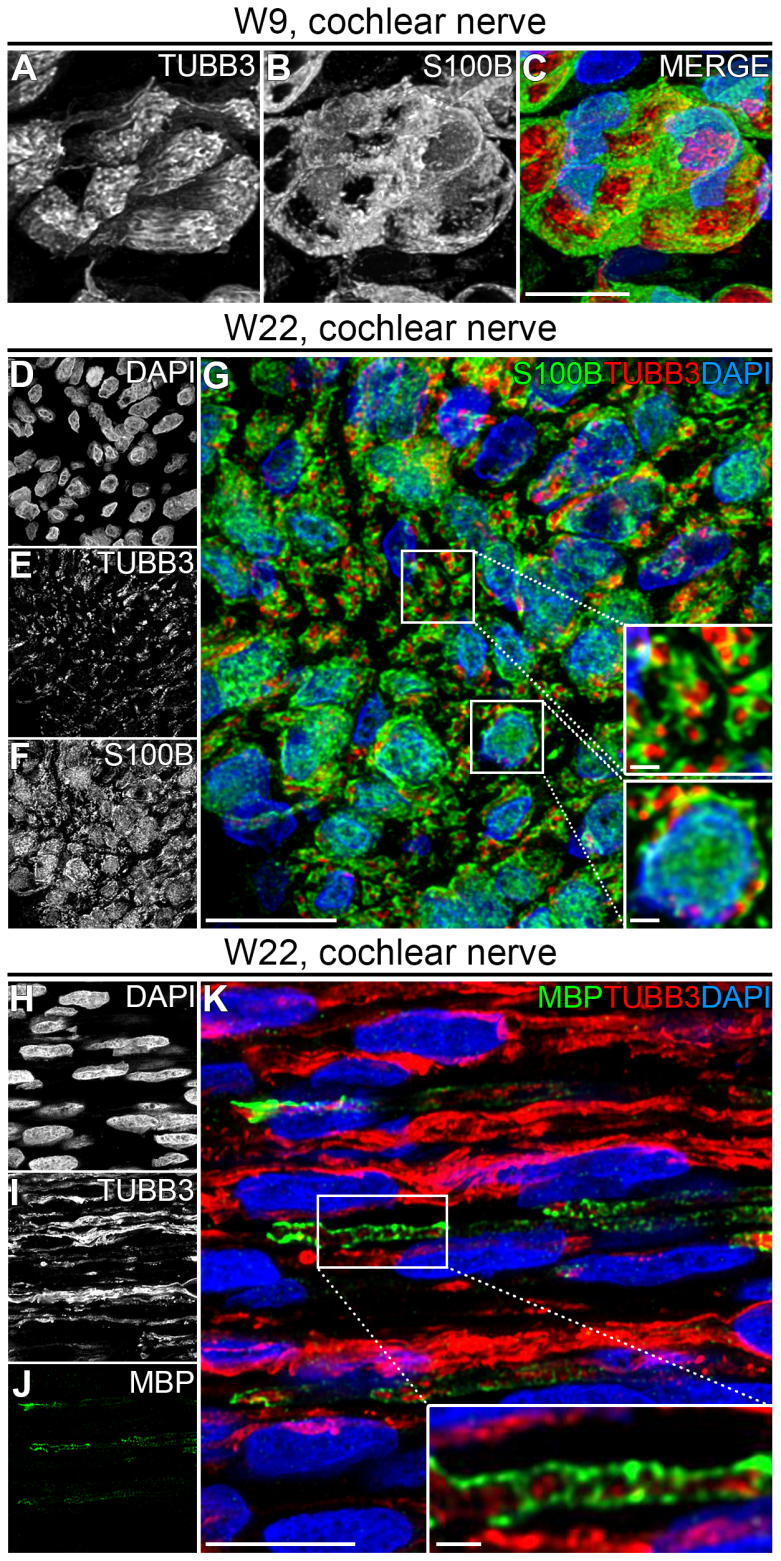
Terminal differentiation of Schwann cells in the cochlear nerve. (A–C) Deconvoluted confocal images of the cochlear nerve at W9 showing TUBB3 (A, red), S100B (B, green), and the merged image with DAPI (C). (D–G) Deconvoluted confocal images of an axial transection of the cochlear nerve at W22 showing DAPI (D), TUBB3 (E), S100B (F) and the merged image (G). The upper inset in G shows a high-magnification of TUBB3+ cochlear nerve fibers each enveloped by S100B+ Schwann cells, the lower inset shows a Remak bundle. (H–K) Deconvoluted confocal images of a sagittal transection of the cochlear nerve at W22 showing DAPI (H), TUBB3 (I), MBP (J) and the merged image (K). The inset shows a high-magnification view of a myelinated nerve fiber. Scale bar = 10 µm or 1 µm (insets in G and K).

**Figure 9:**
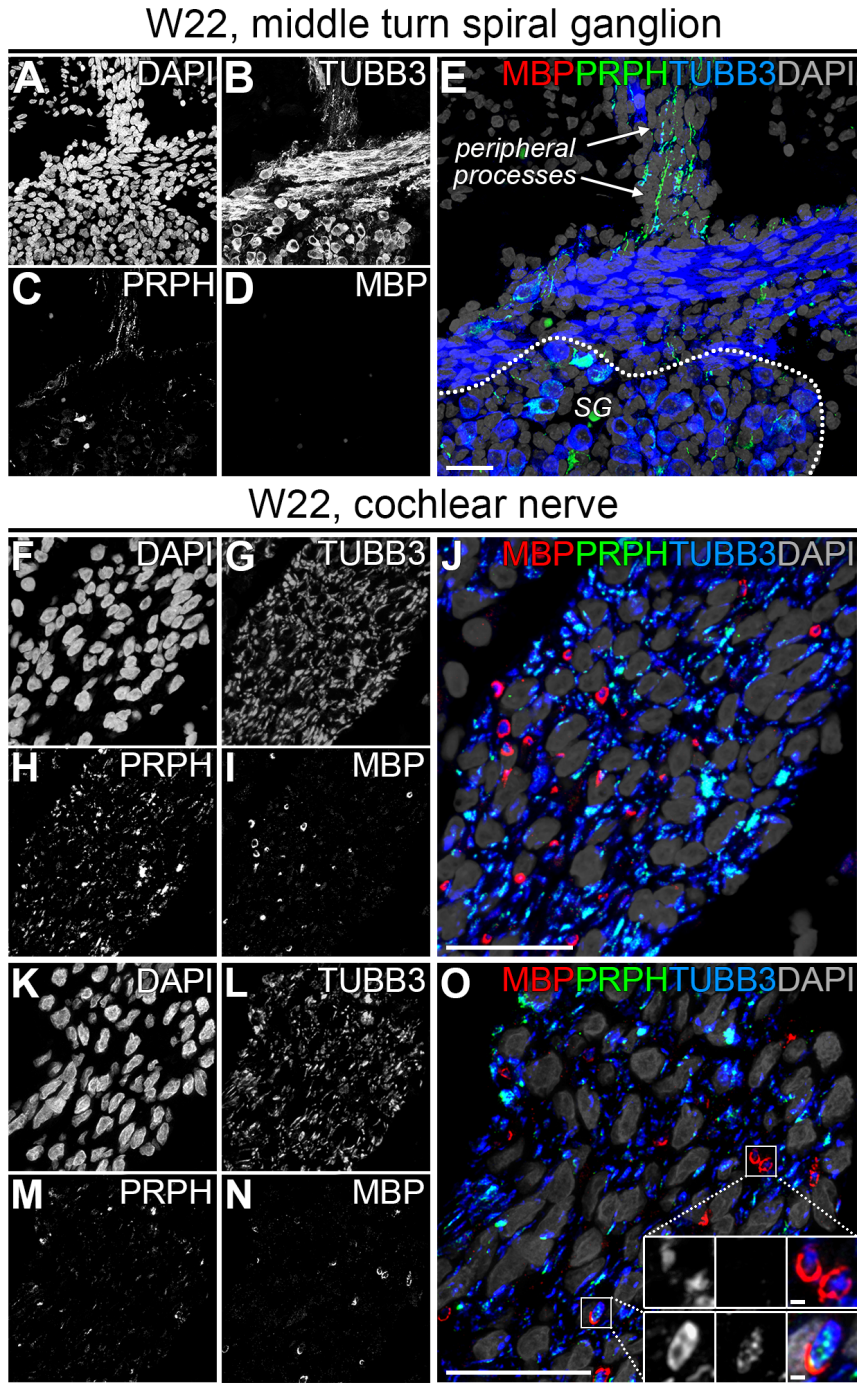
Myelination of spiral ganglion neurons at W22. (A–E) Confocal images of a spiral ganglion in the middle turn of a cochlea at W22 showing DAPI (A), TUBB3 (B), PRPH (C), MBP (D) and the merged image (E). The spiral ganglion is delineated by the dotted line. (F–J) Confocal images of an axial transection of the cochlear nerve at W22 showing DAPI (F), TUBB3 (G), PRPH (H), MBP (I) and the merged image (J). (K–O) Deconvoluted confocal images of an axial transection of the cochlear nerve at W22 showing DAPI (K), TUBB3 (L), PRPH (M), MBP (N) and the merged image (O). Insets show TUBB3 (left), PRPH (middle) and the merge with MBP and DAPI (right) in high-magnifications examples of PRPH−/TUBB3+/MBP+ cochlear nerve fibers (upper inset) and PRPH+/TUBB3+/MBP+ cochlear nerve fibers (lower inset). Scale bar = 20 µm or 1 µm (insets in O).

Immunostaining for S100B and TUBB3 demonstrated that PGCs enveloped and bundled groups of nerve fibers in the CN as early as W9 (Fig. 8A–C). At W22, immunostaining for S100B and TUBB3 revealed that within the CN many individual TUBB3+ cochlear nerve fibers were ensheathed by S100B+ PGCs (Fig. 8D–G and the upper inset in Fig. 8G), suggesting that these cells had differentiated into a promyelinating stage and that radial sorting was completed. In addition, Remak bundles, the final developmental stage of non-myelinating Schwann cells, were observed in the CN (the lower inset in Fig. 8G). To investigate myelination, we analyzed the expression of MBP, a major component of the myelin sheath. We were unable to detect any MBP expression along the peripheral processes, nor within the SG nor within the CN up to W18 (data not shown). At W22, immunostaining for MBP revealed a few MBP+ tubular structures surrounding individual TUBB3+ cochlear nerve fibers, indicating that myelination in the CN had started at least by W22, but not before W18 (Fig. 8H–K). No MBP expression was observed within the SG or along the peripheral processes in any of the cochlear turns at W22 (Fig. 9A–E). To determine if the MBP+ central processes belonged to type I or type II SGNs, we immunostained the CN at W22 for MPB, TUBB3 and PRPH (which selectively labels type II SGNs in the adult human cochlea). In most cases, MBP+ structures were seen along TUBB3+/PRPH− cochlear nerve fibers (Fig. 9F–O and the upper insets in Fig. 9O). However, MBP+ structures along TUBB3+/PRPH+ cochlear nerve fibers could also be observed, albeit only a few (Fig. 9K–O and the lower insets in Fig. 9O).

## Discussion

### On the origin of PGCs in the human cochlea

The assumption that the cells within the cochlea are of dual embryonic origin has been based on classic experiments using spotted salamander larvae [42] and chick-quail chimeras [43]. These experiments indicated that whereas the SGNs are derived from the otic placode, PGCs have a neural crest origin. Recent research has further confirmed the neural crest origin for PGCs in the mouse cochlea [44], [45], suggesting that the PGC cell origin is conserved in vertebrates. The pattern of PGC distribution that we observed in humans at the early gestational stages (W9-12) is in agreement with the hypothesis of a neural crest origin, with neural crest cells or early derivatives migrating along the CN into the human cochlea and populating the SG. Although the developmental phenotypes of the human PGC remain to be fully understood, the expression of S100/S100B consistently marks the transition of Schwann cell precursors into immature Schwann cells in the rat [37], [38]. As S100B was already abundantly expressed at W9, we concluded that the PGCs in the human cochlea at this fetal stage correlate with an immature Schwann cell phenotype. Other differences between Schwann cell precursors and immature Schwann cells in the rat are the expression of glial-fibrillary acidic protein (GFAP), OCT6 and O4 or the presence of a basal lamina [14]. Therefore, in addition to S100B we have investigated the expression of GFAP, however, this marker was found to be expressed by SGNs rather than by the PGCs at W12–W22 (data not shown).

**Figure 10:**
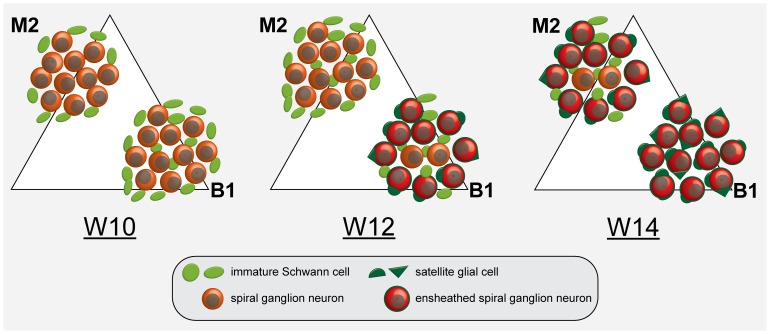
Model of satellite glial cell development in the human spiral ganglion. Satellite glial cell development and the envelopment of the cell bodies of SGNs both occur in a temporal and spatial gradient within the spiral ganglion of the developing human fetal cochlea. W10: Immature Schwann cells (light green) can be found at the edges of the SG in the upper middle turn (M2). In addition, a few Schwann cells are located in between the SGNs (orange) in the lower basal turn. W12: The number of Schwann cells in between the SGNs has greatly increased in M2. Satellite glial cells (dark green) in B1 start to envelop the cell bodies of SGNs (red). The pattern in M2 at W12 resembles that in B1 at W10. W14: All SGN cell bodies in B1 are enveloped by one or more adjacent satellite glial cells. The pattern in M2 resembles that in B1 at W12.

In avian embryos, previous studies have shown that both satellite glial cells and Schwann cells are derived from a S100+ common precursor cell [21], [46]. Based on the onset of association with SGNs, we hypothesize that the satellite glial cell phenotype arises in the human cochlea from W12 onwards, as modelled in Fig. 10. Before this stage, SOX9+/SOX10+/S100B+ PGCs can be found in and around the SG, matching an immature Schwann cell phenotype. Therefore, as in animals (reviewed in [14], [15], [21], [47]), our observations indicate that satellite glial cells, myelinating Schwann cells and non-myelinating Schwann cells in the human cochlea also share a common precursor: the S100B+ immature Schwann cell.

### Schwann cells along the peripheral processes, a role for NGFR?

Interestingly, the PGCs along the peripheral processes and along the central processes in the CN have a different phenotype, as the former express NGFR whereas the latter do not. NGFR is a low-affinity receptor for all neurotrophins and can act alone or in conjunction with Trk-receptors. Among its functions are programmed cell death (apoptosis), cell survival, neurite outgrowth, myelination and Schwann cell migration as well as modulation of synaptic strength [48]–[50]. Although expression of NGFR has been reported in Schwann cells in the adult human cochlea [41], its exact functions in the cochlea remain unknown.

Mouse models have shown that deficiencies or mutations in NGFR are linked to progressive hearing loss, but have also indicated that NGFR is not required for development of hearing (as tested by auditory-evoked brainstem responses) in the first postnatal weeks [49], [51]. In a study exploring the development of neural innervation in the mouse cochlea in *ErbB2* (a Schwann cell receptor involved in survival and myelination) null mutants, the cochlea was found devoid of Schwann cells. Interestingly, in these mutant cochleas, the peripheral processes of SGNs did overshoot the organ of Corti, their target location [52]. The authors speculated that cochlear Schwann cells could normally provide containment signals guiding neurite outgrowth. Other studies in chick and mice also indicate that cochlear Schwann cells form physical corridors and guide SGNs in a reciprocal relationship [53], [54]. Further evidence for this hypothesis comes from a study using a mouse model of peripheral nerve injury that showed strong up-regulation of NGFR by Schwann cells [55]. The resulting increase in NGFR lowered the amount of neurotrophins available to the injured neurons, thereby inhibiting the regenerating axons to penetrate from the dorsal root into the spinal cord.

Our data support the hypothesis of a role for Schwann cells in nerve containment, as we observed a close relationship between NGFR+ Schwann cells and peripheral processes, as early as W9. In addition, Schwann cells did not follow when peripheral processes started to penetrate and explore the cochlear duct epithelium at W12. Whether or not NGFR plays a role in the process of axon guidance within the cochlea remains to be investigated.

### NGFR expression in the developing organ of Corti

In the adult human organ of Corti, expression of NGFR has not been reported [41]. However, studies in mice and rats have detected transient expression of NGFR in pillar cells, disappearing in the first postnatal week, prior to the onset of hearing in these animals [49], [56]–[58]. It is thought that hearing in the human foetus commences at W20 or later [59]; the NGFR expression we detected in the outer pillar cells at W18 support the view of the immaturity of the organ of Corti at this stage. It has been proposed that NGFR expression plays a role in the differentiation or survival of hair and supporting cells and the formation of the tunnel of Corti [56]. Interestingly, we observed the strongest expression of NGFR in the cuticular plate of the outer pillar cell, that is exposed to the lumen of the cochlear duct. A plausible explanation for this observation could be that the regulatory effects of NGFR at this site are mediated by circulating (pro)neurotrophic factors in the fluid of the cochlea acting on the cuticular plate. However, we are unaware of studies investigating the presence of such proteins in the cochlear fluids during development.

### No transient myelination of SGNs by satellite glial cells

In contrast to other mammalian species, the majority of the cell bodies of the SGNs in the adult human are unmyelinated [13], [22], [27], [28]. Transient myelination of SGN cell bodies during human fetal development, from W14 onwards, has been suggested [29]. In the current study, we did not observe any MBP expression within the human SG between W15 and W22. As myelination of SGN cell bodies has also not been observed in human neonates or infants [22], [24], it is unlikely that SGNs undergo a period of transient myelination during human fetal development.

### The onset of myelination within the human cochlea

In an extensive evaluation of the developing human fetal CN, it was reported that myelination of the central processes in the CN starts at W20 [31]. Myelination of the peripheral processes has been observed at W24 [33]. We investigated Schwann cells along peripheral processes up to W22, but did not detect MBP expression in any of the basal and middle turns. However, at W22 we observed MBP in the CN. Together, this shows that the onset of myelination of the CN, at W20, precedes myelination of the peripheral processes, which occurs between W22 and W24.

In the adult human cochlea, the number of type I SGNs correlates well with the number of myelinated nerve fibers within the CN and it is commonly accepted that the unmyelinated fibers represent type II SGNs [25]. As PRPH selectively immunostains the central processes of type II SGNs in the adult human cochlea [60], we examined whether all MBP+ structures surrounded PRPH-negative fibers. Indeed, most of the fibers surrounded by MBP did not express PRPH, but few exceptions were observed. This could indicate that some myelinated cochlear nerve fibers may be of type II SGN origin. However, as all SGNs express PRPH early in development [34], we favor the more plausible alternative explanation that these structures represent (immature) type I SGNs in the process of downregulating PRPH.

In conclusion, our data provide a comprehensive overview of PGC development in the human fetal cochlea, which may add to our understanding of congenital disorders and auditory neuropathies and can be used as a basis to evaluate acquired changes during adult life.
